# Spatial sensing as a strategy for public goods regulation by gut microbes

**DOI:** 10.1093/ismejo/wrae233

**Published:** 2024-11-26

**Authors:** I Hashem, A Zhang, J Van Impe

**Affiliations:** KU Leuven, Chemical Engineering Department, BioTeC & OPTEC, Gebroeders De Smetstraat 1, 9000 Ghent, Belgium; KU Leuven, Chemical Engineering Department, BioTeC & OPTEC, Gebroeders De Smetstraat 1, 9000 Ghent, Belgium; KU Leuven, Chemical Engineering Department, BioTeC & OPTEC, Gebroeders De Smetstraat 1, 9000 Ghent, Belgium

**Keywords:** gut microbiota, microbial regulatory mechanisms, evolution, individual-based modeling

## Abstract

The gut microbiota has evolved in a complex, spatially structured environment, where microbial interactions are shaped by host-secreted molecules. We propose the spatial sensing (SS) hypothesis, which posits that gut microbes regulate costly cooperative traits, such as public goods, based on their proximity to the epithelial layer. First, we explore the evolutionary dynamics and selective pressures that could drive the emergence of SS. We then outline the spatial organization of the gut microbiota, emphasizing diffusion gradients of host-secreted molecules that may serve as positional cues. Depending on the cost–benefit ratio of secreting public goods near the epithelium, we propose two SS regulatory strategies: SS Type I, where production is suppressed in high-cost, low-benefit conditions, and SS Type II, where production is upregulated in nutrient-rich regions where benefits outweigh costs. We evaluate these strategies using an individual-based model simulating microbial competition in the gut environment. Our results show that SS regulation enhances microbial fitness by modulating investment in costly traits according to spatially varying costs and benefits, outperforming constitutive production. Our findings highlight that SS is both beneficial and evolutionarily feasible, as host-secreted molecules create spatial gradients that microbes can exploit for regulatory purposes. By incorporating spatial positioning as an additional regulatory cue, SS could complement quorum sensing (QS) and competition sensing (CS), fine-tuning the expression of costly traits when and where they are most beneficial within the gut environment. This perspective offers new insights into host–microbiota interactions and could inform strategies for modulating gut microbiomes in health and disease.

## Introduction

The gut microbiota has evolved over millions of years in close association with its hosts. With respect to microbe–microbe interactions, evolutionary theory predicts the evolution of microbial regulatory mechanisms that best suit the unique requirements for competing in the gut system. We propose here that gut microbes have evolved spatially dependent regulation mechanisms, linked to their proximity to the epithelial layer of their host. First, we explore the evolutionary dynamics likely to support the emergence of these mechanisms in the gut microbiota and introduce a “spatial sensing” (SS) hypothesis, suggesting that the regulation of costly cooperative traits within the gut is spatially dependent. We then provide an overview of the spatial structures established by the gut microbiota as well as the different types of host-secreted molecules, to shed light on the potential underpinnings of a spatially dependent regulatory strategy. We further explore and evaluate the SS hypothesis through an individual-based model (IbM) of a host-associated microbial community.

## Host–microbiota co-evolution

Understanding the host–microbiome relationship requires considering the evolutionary dynamics of both microbe–microbe and host–microbe interactions. According to the “ecosystem on a leash” model [1], the microbes residing in the gut primarily undergo selection based on their ability to compete and persist within the host. If a bacterial strain provides a fitness advantage to the host but compromises its own growth rate, it will likely be outcompeted by faster-growing strains in the gut community. From an evolutionary standpoint, strains that invest in their own reproduction are expected to be favored by natural selection, regardless of whether they confer a fitness advantage to the host or not. In certain cases, growth-focused strains may even harm their hosts by causing inflammation or invading tissues and the bloodstream, a phenomenon known as “short-sighted evolution” [2], [3]. Meanwhile, hosts are subjected to evolutionary pressure to shape, control, and stabilize their associated microbiota for their own benefit [1]. Host control is achieved through the release of selective nutrients and/or antimicrobials from the epithelial layer, influencing the competition in favor of beneficial strains. The impact of host secretions primarily affects the community’s composition near the epithelial surface. Composition perturbations propagate upward and ultimately influence the outcome of competition within the whole community [4].

Within the densely populated communities of the gut system, bacterial cells of the same genotype can engage in cooperation by producing extracellular enzymes at the cost of individual cells, thereby increasing the availability of resources in their environment. The production of bacteriocins can also be viewed as a cooperative behavior among the cells that produce them, as it benefits the kin at the expense of individual cells. The costly nature of expressing cooperative social traits has driven bacteria to evolve various regulatory mechanisms to limit the expression of such traits until they are most beneficial. One example of such regulatory mechanisms is “competition sensing” (CS), which suggests that bacteria restrain the expression of bacteriocins until they sense ecological stress from competing species [5]. Another commonly observed regulatory mechanism is quorum sensing (QS), where bacteria delay the expression of a costly cooperative trait until they reach a relatively high biomass density, at which the expressed extracellular enzyme or bacteriocin can be most effective [6].

In the gut ecosystem, we argue that the regulation of public goods expression could be influenced by two distinctive features specific to this environment. First, the relative benefits and costs associated with the production of public goods may vary depending on the spatial position of cells along the axis from the epithelium to the lumen. Near the epithelial layer, where competition outcomes can significantly influence population dynamics throughout the community, this balance between costs and benefits becomes particularly crucial. Depending on the specific environmental conditions, it may be advantageous for a strain to either upregulate or downregulate public goods production when positioned close to the epithelial layer. The second feature is the presence of diffusion gradients of host-secreted molecules, which may provide gut inhabitants with cues to infer their position relative to the host epithelial cells. Together, these features suggest that a spatially dependent regulatory mechanism could be both beneficial and feasible in the gut system. We propose this as the SS hypothesis: microbial species in the gastrointestinal tract will adjust the expression of cooperative traits, such as toxin production, based on their proximity to the epithelial layer. To describe how microbial species may spatially regulate the production of public goods, we outline two distinct SS regulation scenarios based on the benefit-to-cost ratio along the axis from the epithelial layer to the lumen. Whereas public goods production requires significant metabolic energy, this cost is relatively lower in nutrient-rich environments, as the energy available for growth is abundant. Conversely, in nutrient-poor environments, where energy is scarce, the metabolic burden of producing non-essential compounds such as public goods becomes higher, making production less beneficial. SS Type I describes a scenario where public goods production is downregulated near the epithelial layer due to high metabolic costs and limited competitive benefits in this region. This may occur when nutrients are more abundant in the lumen than near the epithelial layer, making public goods production energetically costly in the proximity of the epithelium. Hence, cells would benefit by focusing on growth near the epithelial layer and restricting public goods production to regions farther from the epithelium. In contrast, SS Type II refers to a scenario where public goods production is upregulated near the epithelial layer due to a favorable benefit-to-cost ratio in this region. This may occur, for example, when host-secreted nutrients create a nutrient-rich environment near the epithelium. In this case, cells would benefit from upregulating the production of public goods, such as toxins, close to the epithelial layer, as this would give the strain a competitive advantage, enabling it to outcompete others in this nutrient-rich zone. Whether the area near the epithelial layer presents as high-cost or low-cost for a strain will depend on its ability to utilize available carbon sources. Microbes that rely on host secretions, such as mucin, would find the environment near the epithelial layer to be low-cost and high-benefit. In contrast, microbes dependent on lumenal substrates would encounter the same area as high-cost and low-benefit. Ultimately, the benefit-to-cost ratio of producing public goods for a given strain depends on its ability to exploit the resources available in its specific environment.

Whereas QS involves time-dependent gene expression and CS entails the activation of stress responses in a social context, the SS hypothesis suggests that the regulation of costly cooperative traits within the gut may be spatially dependent. The evolution of a regulatory mechanism that capitalizes on spatial information could enable resident strains to survive intense competition in the gut, affording them a distinct advantage over potential migrant species not fully adapted to the gut system.

## Spatial organization of the gut microbiome

Understanding potential spatially dependent regulatory mechanisms of gut microbes necessitates examining the structure of the gut microbiome. Throughout the human gastrointestinal tract (GIT), microbial density and diversity increase, culminating in the densely colonized colon [7]. In the lumen, undigested food particles not only provide attachment sites for microbes but also create a nutrient-rich microenvironment [8]. Specific microbial strains form microclusters on these particles, facilitating the degradation of polysaccharides [9]. The diverse composition of food particles contributes to the heterogeneity of lumen microbiota. Moreover, peristaltic contractions within the lumen promote a high degree of mixing within the microbial community [10]. Predominantly, the lumen microbiome consists of anaerobic bacteria including members of the *Bacteroidaceae* and *Clostridiaceae* families, which specialize in fermenting undigested carbohydrates, producing short-chain fatty acids (SCFA) [7].

The GIT’s epithelial layer folds, forming invaginations known as crypts. Goblet cells within the epithelium secrete mucins, primarily O-glycosylated mucin 2, creating a crosslinked gel structure that acts as a mechanical barrier against luminal contents [11]. In the colon, the mucus layer has a two-gel structure. The inner layer, firm and impermeable, acts as a size-excluding barrier keeping the microbiota at a distance. Due to endogenous proteolysis, the outer layer is loosely structured, enabling colonization by commensal bacteria [11]. The crypts in the distal part of the colon are covered with the dense inner mucus layer and thus are free of microbes. Meanwhile, the crypts in the caecum and proximal colon have been found to be dominated by the phyla *Proteobacteria* and *Deferribacteres* in mice [12]. The crypt-specific core microbiota is protected from the flow of gut content by the crypts and has been found to be enriched for genes encoding stress resistance [12].

The distinct environments of the crypts, mucus layer, lumen, including variations in oxygen levels, nutrient availability, and host secretions, shape specialized microbial communities along the transverse axis of the gut. The colonization of mucosa-associated microbes is likely aided by their ability to metabolize host-derived glycans [13]. In the colon mucus layer, *Bacteroides fragilis*, *Bifidobacteriaceae*, and *Akkermansia muciniphila* are dominant and capable of degrading host-secreted mucin [7]. By feeding on mucin carbohydrates, these mucus-degrading species also offer cooperative benefits for other bacteria, contributing to the overall stability of the mucosa-associated microbiota [7]. For example, *Bifidobacterium dentium* utilizes the by-products of glycan degradation without directly degrading the mucin itself [14]. This species-specific carbohydrate utilization system, termed commensal colonization factors, contributes to the stable colonization of commensal bacteria and resistance against pathogen colonization [15].

The colon’s heterogeneous mucus layer fosters a compartmentalized and spatially specific microbial community. The interface between the dense and loose mucus layers is the most heavily colonized, with colonization density gradually decreasing towards the lumen. In healthy mouse colons, *Bacteroidales* are positioned closer to the epithelium compared with other mucosal residents such as *Bacillota*, indicating a preference for host-secreted mucin over luminal fiber [16]. Furthermore, studies have shown that fiber depletion, which serves as a crucial fermentation substrate for luminal microbiota SCFA production, leads to alterations in the mucosa-associated microbiota’s diversity and spatial organization [17], suggesting that luminal nutrients also shape the mucosa-associated microbiota to some extent.

## Host signaling and public goods regulation in the gut microbiota

**Figure 1:**
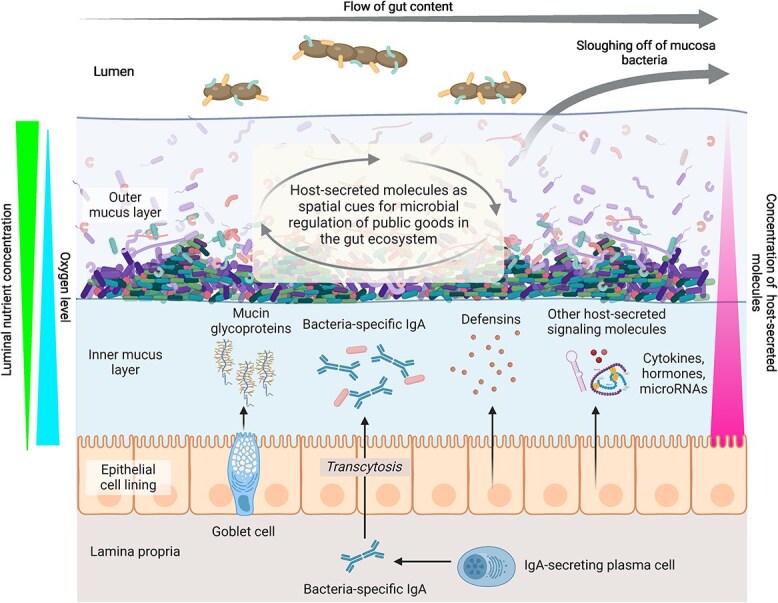
Spatial sensing in the gut microbiota. In the colon, the intestinal mucosal layers consist of a dense inner mucus layer that is impermeable to microbes and a looser outer mucus layer that hosts a diverse community of commensal bacteria. These mucus layers serve as reservoirs for various host-secreted molecules, including mucins, bacteria-specific IgA, defensins, cytokines, hormones, and microRNAs, which can influence bacterial growth positively or negatively. Through diffusion, these molecules build up a concentration gradient. The SS hypothesis suggests that mucosal microbes can sense this gradient, allowing them to infer their position within the gut. Based on this spatial information, microbes can adjust the expression of cooperative traits or the production of public goods, thereby optimizing their competitive advantage within the complex gut ecosystem.

Bacterial growth in the intestinal environment is positively or negatively influenced by an extensive and complex network of bioactive substances within the gut, namely the intestinal eco-active chemosphere [18]. The molecules originated from the host, the diet, and the microbiome function as signaling cues that mediate the ecological interactions of the gut microbiota. Specifically, the host-secreted signaling molecules and metabolites are an important component of the intestinal chemosphere (Table 1), which we hypothesize can serve as cues for the microbiota to sense their spatial position relative to the epithelial layer. The intestinal mucus serves as a reservoir for host-secreted molecules, including hormones, microRNA (miRNA), immune-related molecules, and nutrients [19]. Evidence from *in vitro* cultures and *in vivo* animal models suggests that these host-secreted molecules can directly or indirectly influence microbial metabolism and behavior [20]. Microbiomes are inherently heterogeneous, with spatial variations in microbial composition and activity arising from numerous factors, including local environmental conditions and microbial interactions. Host-secreted molecules further add to this heterogeneity by creating diffusion gradients, with concentrations highest near the epithelial layer and decreasing towards the colony’s edge (Fig. 1). Consequently, microbial cells could infer their position based on these cues and utilize this information to regulate the expression of kin-beneficial traits.

**Table 1:** Overview of host-secreted molecules that can act as cues for mucosal microbes to sense their position relative to the epithelial layer.

**Types of host-secreted molecules**	**Examples**	**Reported functions with respect to the gut microbiota**
**Immune-related molecules**	Secretory IgA	Selectively binding to microbial antigens, limiting pathogen adherence and translocation, promoting immune exclusion [30].
	Cytokines	Affecting the growth and behavior of gut microbes by specifically binding to microbes or by mediating the host immune responses [31–33].
	Defensins	Broad-spectrum antimicrobial peptides, functioning by disrupting the bacterial cell membrane, maintaining microbiota homeostasis and responding to pathogenic signals [34, 35].
**Metabolites**	Mucin	Providing binding sites for specific bacterial adhesins and serving as a source of nutrients for some bacteria [36].
	Fucose	Promoting the growth of commensal strains that easily utilize fucose [1, 26, 37].
**Hormones**	Estrogen and androgen	Affecting the diversity of gut microbiota by functioning as inter-kingdom quorum chemical signaling compounds [38, 39].
	Dehydroepiandrosterone	Modulating the microbiota taxa by potentially serving as a quorum sensing molecule, and by regulating the host immunity [40, 41].
	Insulin	Regulating the growth of the microbiota and functioning as an endogenous QS molecule [42, 43].
	Norepinephrine	Modulating microbial growth by potentially interacting with microbial QseC sensor kinase receptors, and by affecting host immunity and neurotransmitter release [44–46].
**MicroRNAs**	miR-223, miR-1224-5p	Potentially affecting the density and diversity of gut microbiota by altering target gene transcription [34, 47, 48].

The production of public goods, such as extracellular mucin-degrading enzymes secreted by *Bacteroides* species, incurs a physiological cost to the producing species [7]. Therefore, microbes have developed mechanisms to regulate public goods production to maximize their competitive advantage within the complex gut ecosystem. For instance, the production of bacteriocins—antimicrobial peptides that inhibit the growth of bacteriocin-sensitive strains—is a known strategy to increase the fitness of the producer by preventing the colonization of competing bacteria. Bacteriocin production can be triggered by the presence of competing strains (CS) or by reaching a certain colony density (QS) to achieve an effective killing concentration. For example, *Lactobacillus* species, often considered commensal, can regulate bacteriocin production through an auto-induction mechanism mediated by the bacteriocin itself [21].

In the context of gut microbiota, the principles of landscape ecology highlight that spatial heterogeneity can significantly influence metabolic regulation and microbial interactions. Bacteriocin production is influenced by dynamic changes in the micro-environment, including variations in the chemosphere, nutrient availability, temperature, and pH levels. In turn, it has been shown that bacteriocin production can potentially lead to the spatial segregation of bacterial strains, as bacteriocins act as local chemical signals or weapons, fostering competitive exclusion or coexistence. This spatial segregation, by affecting microbial metabolic dynamics, has evolutionary implications as selective pressures imposed by spatial heterogeneity and bacteriocin-mediated competition may drive the evolution of the microbiota [18, 21–24].

Evidence indicates that the mucosa-associated microbiota exhibits a spatially dependent and compartmentalized structure. For the public goods producing strain, inferring its spatial position from gradients of host-secreted signaling molecules could offer advantages in optimizing public goods production strategy. However, very little is known about how the individual members of the microbiota regulate their metabolism based on spatial information. This knowledge gap is partly because traditional sequencing-based technology fails to preserve the spatial information of the gut community [8]. The newly emerging spatially resolved sequencing technology or *in situ* hybridization may resolve the complexities of compartmentalized regulation of public goods pathways of the mucosa-associated microbiota.

In this paper, we aim to use a computational model to provide new insights into how the mucosa-associated microbiota can utilize spatial information to regulate the expression of their costly public goods, thereby increasing their competitiveness in the gut system.

## Model

Individual-based modeling has been extensively applied to study ecological interactions within microbial communities. A previously developed model simulates a two-strain microbial community growing on the host epithelial layer [4]. This model incorporates diffusion gradients of lumen nutrients and host secretions, as well as bacterial reproduction and mechanical shoving, driving the upward motion of cells. The microbial community is constrained by a predefined maximum thickness, beyond which cells slough off into the lumen. This work demonstrates how selective effects from host-secreted nutrients at the colony’s base influence the entire community through positive microbial growth near the epithelial surface. Building on this model, our study shifts focus to microbe–microbe interactions, exploring the impact of spatial regulation of public goods on microbial competition. Specifically, we examine toxin production by a bacteriocin-producing strain $P$ against a sensitive strain $S$, where the strains could represent distinct phenotypes within a species or two separate species competing for the same ecological niche. The model can also approximate more complex communities, where each “strain” represents multiple strains or species sharing a similar phenotype [4]. The competition between the two strains is described by the following set of equations:


(1) \begin{align*} &\frac{dP}{dt} = (1 - f) \mu P, \end{align*}

(2) \begin{align*} &\frac{dS}{dt} = (\mu - K_{T} T)S, \end{align*}

(3) \begin{align*} &\frac{dT}{dt} = \alpha f \mu P - \beta_{T} T, \end{align*}

(4) \begin{align*} &\frac{dN}{dt} = \frac{-1}{Y} \mu (P + S), \end{align*}

(5) \begin{align*} &\mu = \mu_{\textrm{max}} \frac{N}{N + K_{N}}. \end{align*}

$P$ (g bacteria/l) and $S$ (g bacteria/l) represent the biomass densities of the toxin-producing strain and the sensitive strain, respectively. $T$ and $N$ (g/l) are the concentrations of the toxin and the lumen nutrient in the model system. $f$ is the fraction of metabolic energy invested by $P$ in toxin production. $K_{T}$ (l/g toxin/min), $\alpha $ (g toxin/g bacteria), and $\beta _{T}$ (1/min) are the toxin killing rate, the stoichiometric coefficient for toxin production, and its decay rate, respectively. $\mu $ (1/min) and $Y$ are the specific growth rate and the yield coefficient for microbial growth, whereas $\mu _{\textrm{max}}$ (1/min) and $K_{N}$ (g/l) represent the maximum microbial growth rate and the half-saturation constant, with the microbial growth modeled using Monod growth kinetics.

SS regulation is modeled using ${SS}_{\textrm{th}}$, a parameter defining a critical distance from the epithelial layer on the axis from the epithelium to the lumen; in one scenario, cells located below ${SS}_{\textrm{th}}$ do not produce toxins, whereas those above this threshold do so by setting $f = f^{*}$. In the alternative scenario, cells below ${SS}_{\textrm{th}}$ engage in toxin production ($f = f^{*}$), whereas those above it do not. All simulations start with an initialization phase in which the model is seeded with 40 cells of each strain, randomly distributed at the epithelial layer, which grow until reaching the maximum thickness of the community. No toxin production occurs during the initialization phase. After reaching the maximum community thickness, the experiment begins with the fraction of cells belonging to the $P$ strain lying above or below ${SS}_{th}$, depending on the regulation scenario, releasing toxin. The supplementary material contains further parameter details and model implementation specifics.

Our model incorporates several assumptions, as follows [4].

- **Host-secreted molecules are transported to the gut microbiota via diffusion in the mucus layer.** Although the transport mechanism is complex and not fully understood, our model focuses on diffusion, acknowledging that extracellular vesicles (EVs) may also contribute [19]. Diffusion is a recognized key mechanism in various gut models for moving host-secreted molecules through the mucus layer to the microbiota [4, 25].
- **Microbial growth is generally assumed to be positive near the epithelial layer, driving a net upward movement of cells within the densely populated microbial community.** This assumption is based on the well-documented impact of host-secreted nutrients and the mechanical interactions between cells, which facilitate their movement towards the lumen, thereby influencing competitive dynamics throughout the community [4, 26]. The secretion of mucin by the host further supports this upward movement, as it contributes to the physical structure that influences cell positioning and movement within the community [27]. However, it is important to note that this assumption may be context-dependent and could be reversed in the presence of detrimental host factors, such as immune responses or antimicrobial secretions, which would inhibit growth near the epithelial layer.
- **Cells located at the community’s upper layer are less persistent and detach more readily than those near the epithelial layer.** This reflects the natural detachment process in the gut, where cells in the upper mucus layer, being less adherent, are more likely to be sloughed off into the lumen, a phenomenon supported by existing literature [27].

Though our model includes several complex features, such as cell movement driven by mechanical interactions, sloughing of cells into the lumen, and various cellular metabolic processes, it simplifies other aspects of gut ecology to maintain focus. For instance, the model does not consider variability in mucosal cell shedding rates, the presence of additional host-produced deleterious molecules, or the detailed anatomical and pathological features of the gut environment. Although incorporating these factors could add to the model’s realism, they were intentionally excluded to avoid introducing excessive complexity.

We demonstrate two scenarios where spatial regulation of public goods production could benefit a microbial strain in a simple two-strain gut community. In the first scenario, the strains are unable to utilize host-secreted nutrients and must rely solely on lumen-derived nutrients. This creates a high-cost, low-benefit environment near the epithelial layer. Under these conditions, cells would benefit from focusing on growth near the epithelium and producing toxins farther away. This regulation strategy is termed SS Type I. In the second scenario, we examine a high-benefit, low-cost environment near the epithelial layer, where host-secreted nutrients are both abundant and usable by the strains. In this case, cells benefit from upregulating toxin production close to the epithelium and downregulating it farther away. This regulation strategy is referred to as SS Type II.

### Scenario I: high-cost, low-benefit near the epithelial layer

In this scenario, we simulate a high-cost, low-benefit environment near the epithelial layer by assuming that the microbial strains are unable to utilize host-secreted nutrients, forcing it to rely on lumen-derived nutrients that are concentrated farther away. The relative cost of producing public goods depends on both the position of the cell within the community and the availability of nutrients in that location. Cells at the base of the community, close to the epithelial layer, have a longer residence time and exert a significant influence on community composition because they persist longer than cells at the top, which are more likely to be sloughed off. When these basal cells produce diffusible toxins, they face high metabolic costs, but the toxins primarily diffuse upwards, influencing competition in the upper layers, where cells are less critical for long-term community dynamics. As a result, the energy invested by basal cells in producing toxins does not yield substantial competitive advantages, leading to a low benefit-to-cost ratio for toxin production near the epithelial layer. In contrast, cells positioned farther from the epithelial layer, where nutrient availability is higher, can produce toxins that diffuse downward and affect competition in the lower layers without incurring excessive metabolic costs. Therefore, SS Type I regulation strategy, which restricts toxin production to cells farther from the epithelial layer, proves advantageous in this scenario.

**Figure 2:**
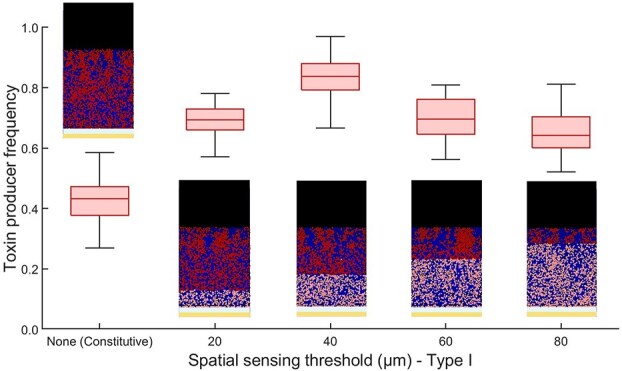
High-cost, low-benefit scenario near the epithelial layer: box plot showing the final frequency of a toxin-producing strain employing SS Type I regulation, where costly public good production occurs only in cells above the ${SS}_{th}$ threshold. The figure displays the final frequency of the toxin-producing strain (red) competing against a sensitive strain (blue) across different ${SS}_{th}$ thresholds for SS Type I regulation, alongside the case of constitutive production. Cells engaging in public good production are highlighted in dark red. In this scenario, where the relative cost of public good production is higher near the epithelial layer, the results indicate that SS Type I regulation is beneficial, providing a competitive advantage over constitutive production.

The fitness of the SS Type I regulated strain, with fitness defined as the strain’s proportion within the community at the end of the experiment, was evaluated against varying thresholds of ${SS}_{th}$: $20$, $40$, $60$, and $80$  $\mu \textrm{m}$, as well as a scenario of constitutive production without regulation (Fig. 2). In the absence of regulation, one might expect the red strain to outcompete its opponent if the toxin it produces is highly lethal and inexpensive to produce. However, because the production of public goods is typically metabolically costly and reduces the growth rate of the producing strain, the outcome of the competition ultimately depends on whether the toxin’s benefits outweigh its production costs. The blue, sensitive strain is observed to outgrow the red toxin-producing strain. This happens because the toxin production, while hindering its opponent’s growth, reduces the red strain’s own growth rate. As a result, the red strain is gradually outcompeted, as the toxin’s lethal effect does not offset its slower growth rate.

With the introduction of SS Type I regulation, the fitness of the toxin-producing strain is significantly enhanced compared with the constitutive production scenario. Maximum fitness is achieved at ${SS}_{th} = 40$  $\mu \textrm{m}$. Beyond this point, as ${SS}_{th}$ increases, so does the thickness of the layer of the red strain dedicated to rapid growth, which results in a decrease in the fraction of cells producing the toxin. Consequently, the fitness of the toxin-producing strain gradually decreases beyond $40$  $\mu \textrm{m}$, approaching the fitness level of its non-toxin-producing counterpart. Overall, SS Type I regulation strategy is beneficial in this high-cost, low-benefit scenario as it restricts toxin production to cells farther from the epithelial layer, optimizing fitness by prioritizing growth near the epithelial layer. In contrast, when testing the SS Type II regulation strategy and stochastic toxin production strategies in this set-up, both result in lower fitness due to a less optimal spatial distribution of toxin-producing cells (see Figures S1 and S2 in the supplementary material).

### Scenario II: high-benefit, low-cost near the epithelial layer

In this scenario, the strains are able to utilize epithelial secretions, with epithelial nutrient production set to 2 mg/l/min, making it the primary driver of microbial growth, while lumen nutrient concentration is reduced to 0.1 g/l. This creates a nutrient-rich environment near the epithelial layer and a nutrient-poor region farther away. As a result, cells near the epithelial layer face lower relative costs for producing public goods, allowing them to benefit from upregulating toxin production to outcompete rivals and secure resources. Conversely, cells farther from the epithelial layer, where nutrient availability is lower, experience higher relative costs for public goods production. In this nutrient-poor environment, these cells would benefit from prioritizing growth over toxin production, as the metabolic burden of producing toxins outweighs the competitive advantage.

**Figure 3:**
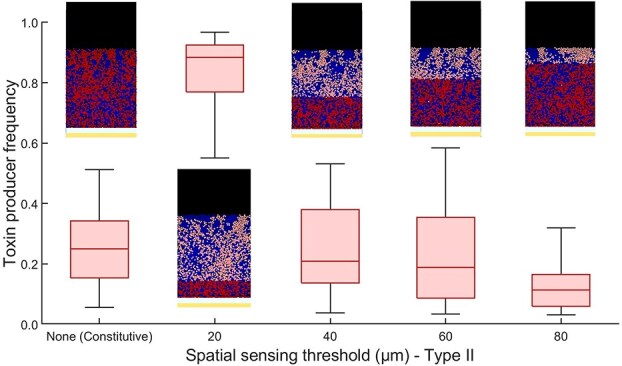
High-benefit, low-cost scenario near the epithelial layer: box plot showing the final frequency of a toxin-producing strain employing SS Type II regulation, where public good production occurs only in cells below the ${SS}_{th}$ threshold. The toxin-producing strain (red) competes against a sensitive strain (blue) across various ${SS}_{th}$ thresholds for SS Type II regulation, alongside the case of constitutive production. Cells producing the public good are highlighted in dark red. In this scenario, the highest fitness for the toxin-producing strain is achieved at ${SS}_{th} = 20\, \mu \textrm{m}$, where cells near the nutrient-rich epithelium upregulate toxin production, while those farther away prioritize growth. The results highlight that the spatial regulation of public good production is driven by the benefit-to-cost ratio along the axis from the epithelium to the lumen.

The SS Type II regulation strategy, which promotes toxin production close to the epithelium, leads to the highest fitness for the toxin-producing strain at ${SS}_{th} = 20$  $\mu \textrm{m}$ (Fig. 3). In this case, cells below the threshold upregulate toxin production in the nutrient-rich areas near the epithelium, while cells above the threshold, in the nutrient-scarce areas, focus on growth rather than investing in toxin production. As the benefit-to-cost ratio of toxin production decreases farther from the epithelial layer, the fitness of the toxin-producing strain drops as ${SS}_{th}$ increases. These results show that the spatial regulation of public goods production is primarily determined by the benefit-to-cost ratio along the axis from the epithelium to the lumen. Although this ratio is influenced by factors such as the spatial distribution of nutrients, the nature of epithelial secretions, and the diffusivity of public goods, further experimental and theoretical investigations will be needed to fully understand the interplay between these factors.

## Concluding remarks

We employed an IbM to simulate microbe–microbe competition within the gut, demonstrating the benefits of spatial regulation for secreting costly public goods. SS regulation was modeled by adjusting toxin release based on a critical distance from the epithelial cells, defined by the parameter ${SS}_{th}$. This would correspond to a regulatory pathway that modulates toxin production in response to the concentration of a host-produced molecule, serving as a proxy for the position of a bacterial cell. This approach was chosen to simplify the modeling process, with parameters such as the rate of host molecule release, its diffusion rate, and the concentration threshold for regulation being encapsulated into the single parameter ${SS}_{th}$. Also, in the current model, host-secreted molecules serve as cues for microbial SS. *In vivo*, these host-derived molecules can exert both positive (e.g. mucin, fucose) and negative impacts (e.g. IgA, defensins) on bacterial growth. The interplay of these factors imposes selective pressures on microbial populations and alters the benefit-to-cost ratio of public goods production. Whereas the current model incorporates host-secreted nutrients as beneficial factors promoting microbial growth, it could be further extended to include host-derived inhibitory factors, such as immune effectors, to better understand the impact of potential microbial SS regulation in different host environments.

Whereas the SS hypothesis has been primarily investigated within the context of the gut microbiome in this paper, its principles may extend to other host-associated microbiomes, such as those of the skin or lung [28, 29]. These host environments also exhibit spatially structured microbial communities, with varying gradients of host-secreted factors and distinct physical barriers. However, given the unique layered structure across the transverse axis of the gut, we chose to first examine the SS hypothesis within the gut microbiota. Future studies could investigate whether similar SS regulation could increase the fitness of a focal strain by utilizing spatial cues to regulate the expression of cooperative traits.

Why would hosts evolve to send signals that assist their microbial residents in navigating microbe–microbe interactions in the first place? There are two potential explanations. First, it is likely that host molecules used by microbes to infer their spatial position did not evolve specifically for this purpose. Hosts are selected based on their ability to shape their microbiome to be most beneficial to them [1]. Host regulation can occur through the release of selective nutrients, toxins, and signaling molecules to steer the community composition towards more favorable host–microbe interactions. This wealth of molecules could serve as cues for the residing strains that they can capitalize on in navigating microbe–microbe interactions. Additionally, the host may also extract a direct benefit from optimizing these strains to be highly adapted to compete within the gut system, as this makes the gut more resistant to invasion by alien, potentially pathogenic species.

Though we have focused on host-secreted molecules as a proxy for public goods regulation within the gut microbiota, spatial information in the gut is likely shaped by a broader range of signals, including chemical and biochemical cues from both the host and microbes, as well as potential influences from diet and food components. Together, these factors add further complexity to the regulatory landscape of gut inhabitants [18, 22].

With respect to host-secreted molecules, several types of secretions could serve as spatial cues within the gut. These include hormones such as insulin and norepinephrine, which act as inter-kingdom signals and typically have higher concentrations near the epithelial layer. Similarly, host-derived microRNAs (miRNAs), such as miR-223, might modulate microbial gene expression by triggering epigenetic changes that influence the expression of social phenotypes in a spatial manner depending on their concentrations. Additionally, immune-related molecules such as cytokines and defensins, along with metabolites such as fucose released by host cells, could help microbes infer their position within the gut. The spatial information provided by one or more of these mechanisms can be further integrated with other signals within the microbiome, such as self-produced quorum molecules, quorum molecules from competing species, and nutrient concentrations, to fine-tune the regulation and optimization of social phenotypes. SS can complement QS by adding a spatial dimension to microbial regulation. While QS coordinates group behaviors based on population density, SS could enable microbes to further optimize their behavior according to their spatial position. For example, SS can localize the expression of beneficial public goods to areas where the benefit-to-cost ratio is highest. Similarly, SS information can be combined with cues related to the social composition of the microbiome, enhancing CS by providing the spatial context necessary for microbes to deploy competitive strategies more effectively. For instance, in situations where toxin production far from the epithelial layer is less advantageous, SS can ensure that the production of antimicrobials in response to competition from other species is upregulated near the epithelial layer and downregulated farther from it. In short, integrating spatial information with existing social sensing systems helps microbes better adapt to the gut environment.

Finally, efforts to understand gut ecology have primarily focused on the study of how microbes, especially pathogens, can affect the host’s health. Yet, it is equally crucial to explore the adaptive mechanisms bacteria have evolved to compete within the gut ecosystem. Such insights are essential not only for understanding how pathogens establish themselves but also for developing strategies to enhance the growth of probiotic species and promote gut health.

## Supplementary Material

Manuscript-Supplementary-Material_wrae233

## Data Availability

The code that supports the findings of this study is openly available on GitLab at https://gitlab.kuleuven.be/u0101487/spatialsensing.
