# Supplementary material for: Spatial sensing as a strategy for public goods regulation by gut microbes
Source: ISME J. 2024 Nov 26;19(1):wrae233. doi: 10.1093/ismejo/wrae233 (PMC12551461; doi:10.1093/ismejo/wrae233)
Supplement: Manuscript-Supplementary-Material_wrae233 [file manuscript-supplementary-material_wrae233.pdf]

Supplementary Material

The nominal values of all parameters can be found in Table 1.

Parameter	Units	Value
$\mu_{\max}$: Maximum growth rate	min^{-1}	0.0088
K_N: Monod half-saturation constant	g l^{-1}	0.000539
$Y_{\max}$: Apparent yield at $\mu_{\max}$, corrected for maintenance	gDW/g nutrient	0.45
f: Toxin investment fraction	NA	0.2
K_T: Toxin killing rate	l g toxin^{-1} min^{-1}	0.4
α: Toxin stoichiometric coefficient	g toxin g bacteria^{-1}	4
β_T: Toxin decay rate	min^{-1}	0.00166
Lumen nutrient concentration	g l^{-1} min^{-1}	20 (Sc. I)<br>0.1 (Sc. II)
$V_{\mathrm{d,min}}$: Minimum volume at division	l	2.6e-15
D_i: Diffusion coefficient of substance i	μm^2 min^{-1}	815
Diffusion coefficient of toxin	μm^2 min^{-1}	$D = 815$ (Sc. I)<br>$D \times 10^{-1}$ (Sc. II)
ρ: Density of cellular biomass	gDW l^{-1}	290.0
k: Intercellular distance factor in cell shoving mechanism	NA	1.3
CV: Coefficient of variance of specific growth rate	NA	0.1

Table 1: Parameter values and definitions for the model (Kreft, 2004; Tack et al., 2015).

The model is implemented using MICRODIMS, an in-house Individual-based Modeling (IbM) platform dedicated to simulating bacterial growth (Verhulst et al., 2011; Tack et al., 2015, 2017), following the design principles of other IbM toolkits in literature (Picioreanu et al., 1998; Kreft et al., 2001; Xavier and Foster, 2007; Mitri et al., 2011). MICRODIMS is developed using the Repast Simphony toolkit (North et al., 2013). Cells are represented as individual entities that grow and reproduce on the epithelial layer of the host. Mechanical collisions between cells are modeled using a relaxation algorithm adapted from (Kreft et al., 2001). The diffusion of the nutrient and the toxin is modeled using Fick's diffusion law and numerically solved using a discretized Forward-Time Central-Space (FTCS) algorithm. A Neumann boundary condition applies at the epithelial layer, setting the concentration gradients of all chemical species to zero at the lower boundary of the simulation, other than the gut secretions. At the upper boundary, a Dirichlet boundary condition is imposed with the nutrient concentration in the lumen being fixed. The dimensions of the environment grid are set to 200×160 μm. The slough-off distance, representing the maximum

thickness of the community, is set to $100\mu m$. All the simulations are run for 140 hours.

Additional Figures

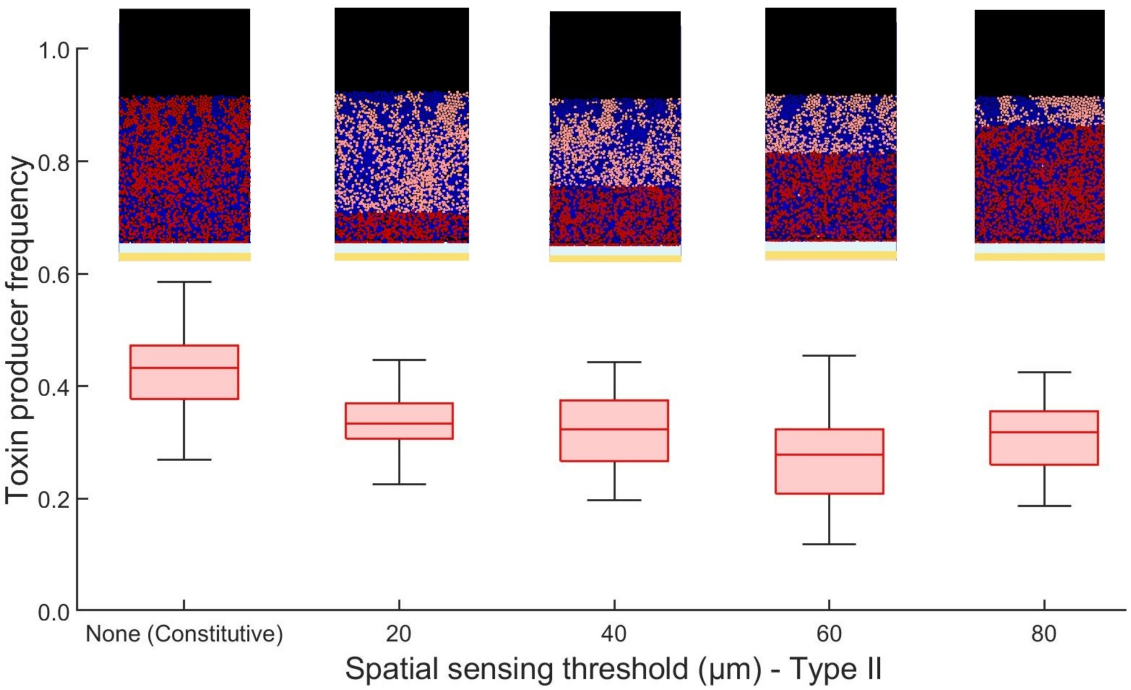

Figure 1: High-cost, low-benefit scenario near the epithelial layer: Box plot showing the final frequency of a toxin-producing strain employing SS Type II regulation, where costly public good production is upregulated near the epithelial layer. The figure displays the final frequency of the toxin-producing strain (red) competing against a sensitive strain (blue) across different SS_{th} thresholds for SS Type II regulation, alongside the case of constitutive (unregulated) production. Cells engaging in public good production are highlighted in dark red. In this scenario, where the relative cost of public good production is higher near the epithelial layer, the results indicate that SS Type II regulation is counterproductive, offering no advantage and potentially reducing the strain's competitive fitness.

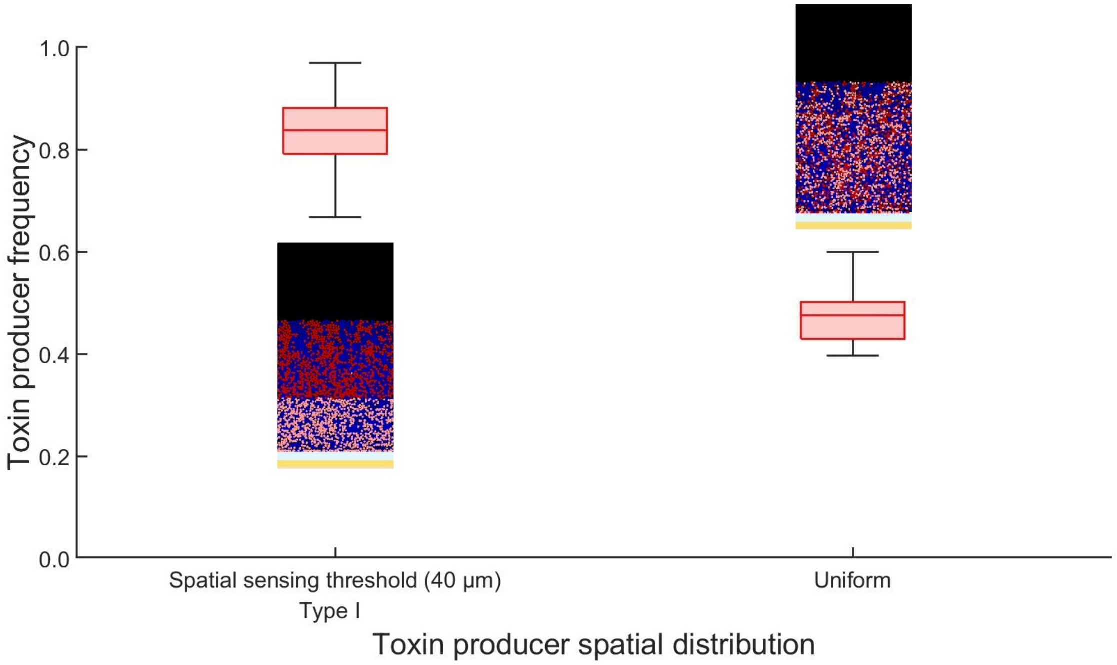

Figure 2: High-cost, low-benefit scenario near the epithelial layer: Box plot showing the final frequency of a toxin-producing strain employing SS Type I regulation, with the regulatory threshold SS_{th} set at 40 µm. The figure compares this to the final frequency of the toxin-producing strain when production is spatially random and uniformly distributed throughout the community, while maintaining the same proportion of toxin-producing cells as in the first case. The toxin-producing strain is depicted in red, and the sensitive strain in blue. In this scenario, where the relative cost of public good production is higher near the epithelial layer, the results demonstrate that the advantage gained by SS Type I regulation is due to a more optimal spatial distribution of toxin-producing cells, where toxin production is downregulated in the costly region near the epithelial layer and upregulated further from it.

References

Kreft, J.-U., 2004. Biofilms promote altruism. Microbiology 150 (8), 2751–2760.

Kreft, J.-U., Picioreanu, C., Wimpenny, J. W., van Loosdrecht, M. C., 2001. Individual-based modelling of biofilms. Microbiology 147 (11), 2897–2912.

Mitri, S., Xavier, J. B., Foster, K. R., 2011. Social evolution in multispecies biofilms. Proceedings of the National Academy of Sciences 108 (Supplement 2), 10839–10846.

North, M. J., Collier, N. T., Ozik, J., Tatara, E. R., Macal, C. M., Bragen, M., Sydelko, P., 2013. Complex adaptive systems modeling with repast simphony. Complex adaptive systems modeling 1 (1), 3.

Picioreanu, C., Van Loosdrecht, M. C., Heijnen, J. J., 1998. Mathematical modeling of biofilm structure with a hybrid differential-discrete cellular automaton approach. Biotechnology and bioengineering 58 (1), 101–116.

Tack, I. L., Logist, F., Fernández, E. N., Van Impe, J. F., 2015. An individual-based modeling approach to simulate the effects of cellular nutrient competition on escherichia coli k-12 mg1655 colony behavior and interactions in aerobic structured food systems. Food microbiology 45, 179–188.

Tack, I. L., Nimmegeers, P., Akkermans, S., Hashem, I., Van Impe, J. F., 2017. Simulation of escherichia coli dynamics in biofilms and submerged colonies with an individual-based model including metabolic network information. Frontiers in microbiology 8, 2509.

Verhulst, A., Cappuyns, A., Van Derlinden, E., Bernaerts, K., Van Impe, J., 2011. Analysis of the lag phase to exponential growth transition by incorporating inoculum characteristics. Food microbiology 28 (4), 656–666.

Xavier, J. B., Foster, K. R., 2007. Cooperation and conflict in microbial biofilms. Proceedings of the National Academy of Sciences 104 (3), 876–881.
